# The Predictive Role of Modified Early Warning Score in 174 Hematological Patients at the Point of Transfer to the Intensive Care Unit

**DOI:** 10.3390/jcm10204766

**Published:** 2021-10-18

**Authors:** Catalin Constantinescu, Sergiu Pasca, Sabina Iluta, Grigore Gafencu, Maria Santa, Ciprian Jitaru, Patric Teodorescu, Delia Dima, Mihnea Zdrenghea, Ciprian Tomuleasa

**Affiliations:** 1 Department of Hematology, Iuliu Hatieganu University of Medicine and Pharmacy, 400349 Cluj Napoca, Romania; constantinescu.catalin@ymail.com (C.C.); pasca.sergiu123@gmail.com (S.P.); iluta.sabina@yahoo.com (S.I.); mariia.elena@yahoo.com (M.S.); Ciprianjitaru.jitaru@gmail.com (C.J.); patric_te@yahoo.com (P.T.); deli_dima@yahoo.com (D.D.); m.zdrenghea@yahoo.com (M.Z.); 2 Department of Anesthesia and Intensive Care, Iuliu Hatieganu University of Medicine and Pharmacy, 400349 Cluj Napoca, Romania; 3 Medfuture Research Center for Advanced Medicine, Iuliu Hatieganu University of Medicine and Pharmacy, 400337 Cluj Napoca, Romania; 4 Intensive Care Unit, Emergency Hospital, 400006 Cluj Napoca, Romania; 5 Department of Hematology, Ion Chiricuta Cancer Center, 400015 Cluj Napoca, Romania; 6 MRC Molecular Haematology Unit—The MRC Weatherall Institute of Molecular Medicine, University of Oxford, Oxford OX3 9DS, UK; grigore.gafencu@rdm.ox.ac.uk; 7 Division of Hematologic Malignancy, Department of Oncology, Johns Hopkins University, Baltimore, MD 21211, USA

**Keywords:** modified early warning score, hematology, intensive care, prognosis

## Abstract

Introduction: The examination of vital signs and their changes during illness can alert physicians to possible impending deterioration and organ dysfunction. The Modified Early Warning Score (MEWS) is used worldwide as a track and trigger system that can help to identify patients at risk of critical illness. Thus, the current study aimed to assess the ability of MEWS to predict the mortality of hematologic patients at the point of transfer from the ward to the intensive care unit (ICU). Materials and Methods: The present study was retrospective, longitudinal, and observational, conducted at an oncology hospital in the city of Cluj-Napoca, Romania. We included 174 patients with hematological disorders transferred from the ward to the ICU between the 1st of January 2018 and the 1st of May 2020. We assessed the MEWS at the moment of admission in these patients in the ICU. The accuracy of MEWS in predicting mortality was assessed via the area under the receiver operating characteristic curves (AUC), and sensitivity, specificity, and hazard ratio (HR) were calculated for different MEWS cutoffs. MEWS values considering the status at discharge and frequency of death by MEWS were also analyzed. Results: We calculated MEWS values considering the status at discharge (*p* < 0.0001), and we assessed the frequency of death by MEWS. We also calculated the hazard ratio (HR) of death depending on the selected MEWS cutoff. The best cutoff point was found to be ≥6, with an accuracy of 0.667, sensitivity of 0.675, specificity of 0.646, and AUC of 0.731. Patients with higher MEWS had a higher probability of mortality. Conclusion: The MEWS and cutoff points were determined on a sample of hematologic patients at the moment of admission to the ICU. The final aim is to encourage physicians to use these scores to improve awareness of organ failure to admit patients to the ICU sooner and limit overall morbidity and mortality. The presence of an ICU physician on ward rounds might help in reducing the timeframe of access to a high-dependency unit (HDU) or ICU. An extension of these scores outside hematologic patients or considering hematologic patients outside ICU must be further studied.

## 1. Introduction

As the field of hematology continuously gains new diagnostic, prognostic, and therapeutic tools, hematologic diseases become better classified and more appropriately treated, leading to the patients gaining better overall survival (OS) and relapse-free survival (RFS). Nonetheless, some patients still reach conditions that mean they require transfer to the intensive care unit (ICU). The population of hematologic patients represents a frail category of patients, frequently being older compared to the general population and being immunosuppressed, thus bringing further difficulties to their management. Because of this, the recognition of signs and symptoms that are associated with organ dysfunction is a highly important clinical skill [1].

The most common premonitory signs of clinical deterioration that occur over several hours and are often missed by ward personnel are hypotension and a fall in the Glasgow Coma Scale/Score (GCS) [2]. These and other changes in the clinical profile of a patient have been used to form track and trigger systems such as the Modified Early Warning Score (MEWS) [3], which is one of the basic algorithms used for the initial evaluation of patients on wards. This scoring system has already been used in hospitals worldwide and has been shown to be able to predict various patient outcomes, including its use as an independent predictor of mortality after in-hospital cardiac arrest [4]. Young et al. showed that the implementation of a protocol incorporating the MEWS or other derivative scores to trigger a patient evaluation and transfer to the ICU was able to reduce code calls by approximately 50% and preventable codes by nearly 75% [5]. Additionally, an important asset of the MEWS is that it offers a simple system that minimizes the risk of forgetting to evaluate some of the most important vital parameters, such as level of consciousness, respiratory rate, systolic blood pressure, heart rate, temperature, and urine output [6]. Table 1 further describes the MEWS.

Regarding MEWS classification, a patient is expected to require a high-dependency unit (HDU) care if they score 3 in a single component, regardless of the final calculated result. Scores equal to or above 5 are statistically linked to an increased likelihood of death or admission to an intensive care unit [3].

The aim of this study was to assess the capacity of the MEWS to predict the mortality of hematologic patients at the point of transfer to the ICU. Besides this, the prognostication of the MEWS may be used as a tool to further sensitize ward staff to the failure of critical illness recognition and improve future training in the clinical field.

## 2. Materials and Methods

The present study was longitudinal, retrospective, and observational.

### 2.1. Patients

The current study was performed at the Ion Chiricuta Cancer Center (IOCN) in the city of Cluj-Napoca, Romania. The retrospective data analysis included 174 patients with hematological disorders transferred from the hematology department to the ICU of the same oncology institute. We included patients that were transferred between 1 January 2018 and 1 May 2020. This study was conducted in accordance with the Declaration of Helsinki and received the approval of the Ethics Committee of IOCN (ethics approval code: 78/24.03.2021).

### 2.2. Study Definitions

We assessed the MEWS at the moment of admission of these patients in the ICU and we collected the following variables: age, gender, time spent in ICU, time spent in hospital before ICU, hematological diagnosis, respiratory rate, heart rate, systolic blood pressure, temperature, urinary output, Glasgow Coma Score (GCS), death at discharge from ICU, and calculated MEWS. All the mentioned variables, except time spent in ICU, death at discharge from ICU, and urinary output were considered at the time the patient was transferred.

If a patient was intubated at the moment of transfer by the rapid response team, the respiratory rate MEWS at admission into the ICU received 3 points (maximum), as it was no longer possible to appreciate the natural respiratory rate of these patients. The patients were intubated either because of severe respiratory failure or cardiac arrest. Additionally, intubation is known to be associated with a severe outcome [7].

The urinary output was assessed per 24 h, with low urine output being <400 mL/24 h.

### 2.3. Data Analysis

Data analysis was performed using R 3.5.1. Categorical variables were represented as an absolute number (percent). Fisher’s test was used to analyze contingency tables. The normality of the distribution was assessed using the Shapiro–Wilk test and histogram visualization. Non-normally distributed data were represented using the median (quartile 1, quartile 2). Fisher’s test was used to determine the OR and between the MEWS cutoff and the discharge status. The package cutpoint was used to calculate the cutoff points, with the method chosen being “maximize_metric” and the metric chosen being “youden”. A *p*-value under 0.05 was considered statistically significant.

## 3. Results

In the current study, we included 174 patients with hematological disorders transferred between the 1st of January 2018 and the 1st of May 2020 from the hematology department to the ICU of the same oncology institute. Of those, 126 (72.4%) were dead at discharge, while 48 (27.6%) were alive at discharge from the ICU. The general characteristics of these patients are presented in Table 2.

**Figure 1:**
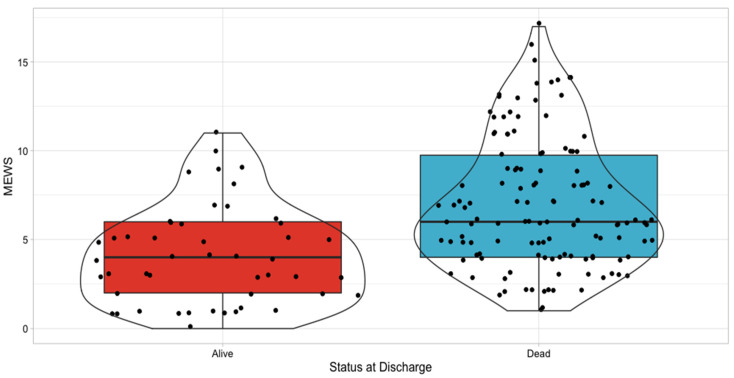
MEWS values considering the status at discharge.

Figure 1 represents the calculated MEWS values considering the status at discharge, with the results being statistically significant (*p* < 0.0001).

**Figure 2:**
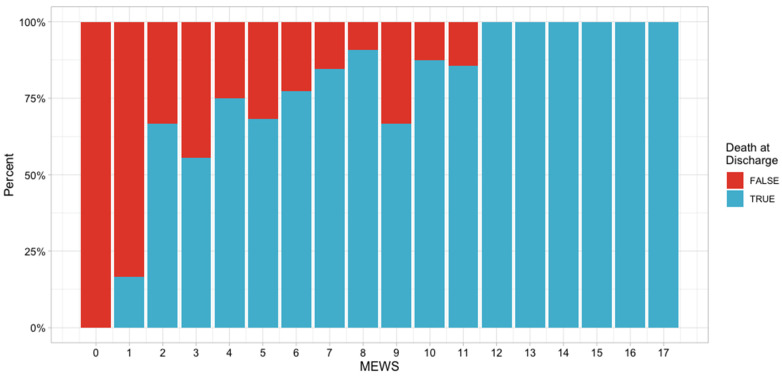
Frequency of death by MEWS score.

Furthermore, we assessed the frequency of death via the MEWS score using a graphical representation (Figure 2).

**Figure 3:**
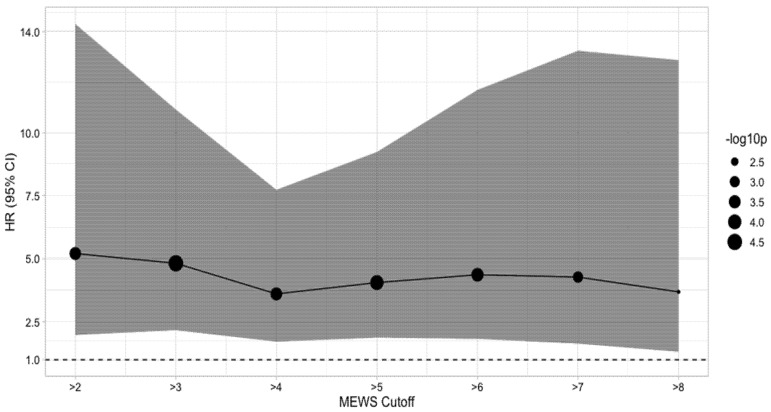
HR of death depending on the selected MEWS cutoff. HR: hazard ratio; CI: Confidence intervals.

We also calculated the hazard ratio (HR) of death depending on the selected MEWS cutoff. The best cutoff point is ≥6, with an accuracy of 0.667, sensitivity of 0.675, specificity of 0.646, and AUC of 0.731 (Figure 3).

## 4. Discussion

National Institute of Clinical Excellence (NICE) guidelines recommend the use of the physiological track and trigger systems to better identify the patients who might benefit from ICU transfer [8]. For example, MEWS can predict ICU mortality in the case of patients admitted with renal sepsis [9], of patients intubated with COVID-19 pneumonia [10], of elderly patients [11], in patients presenting upper gastrointestinal bleeding [12], and other similar situations. Because of the validation of MEWS on several different populations of patients from the ICU setting, it can be said that this score is widely applicable and should be assessed in even more situations as itself or as its different components. Considering hematologic patients, it is more important to determine their prognosis once in the ICU, as Cooksley et al. showed that patients with a hematological malignancy are more likely to be admitted to ICU [13]. Moreover, others determined that cancer patients have a high probability of dying [14]. Others have investigated the usefulness of a set of 10 variables taken at 72 h from the admission in predicting ICU mortality in the case of cancer patients showing an AUC of 0.820 [15]. In our study, the best model was represented by cutoff point ≥6 with an AUC of 0.731, thus showing a similar predictive capacity of mortality in the hematologic population. Periodic simulation-based training of nurses and doctors with the MEWS can improve their knowledge and confidence to promptly react in different clinical scenarios [16].

The main strength of this study is the correlation between a high MEWS value and death in these patients, which can help understand more about the importance of daily rigorous organ assessment. Delaying proper access to more complex monitoring methods and treatment can generate a snowball effect. The presence of an ICU physician on ward rounds might help in reducing the timeframe of access to HDU or ICU due to different clinical experiences.

Regarding the limitations of our study, the most obvious is the high mortality rate in this population. The main reasons for this are the pathology concerned and the high MEWS values at admission. A study by Contejean A et al. concluded an overall mortality rate of 42% due to acute respiratory failure in patients with hematological malignancies [17]. Even though over the last few years, hospital deaths decreased by 30% across the USA (54.6% in 1999 to 38.2% in 2015), there is still a disparity between states, for example, New York had a hospital death rate of 61.6% while Utah had 32.5% [18]. The second limitation of the study is that the MEWS was calculated at the moment of admission in the ICU, not on the ward, with the possibility that the patient further deteriorated between the transfer and admission point in time, as such, gaining more points to the final calculated score. The rapid response team operates between the ward and the ICU and assesses the patient with the ABC approach and Advanced Life Support (ALS) guidelines in order to achieve respiratory and hemodynamic stability during transfer. Nonetheless, rapid clinical deterioration is frequent once organs begin to shut down, meaning there should be a trend towards earlier HDU/ICU admission where continuous and complex monitoring is available.

Different institutions may use different modifications of the MEWS but incorporate the same vital signs. Normal respiratory rate (RR) for an adult person at rest ranges from 12 to 18 breaths per minute, so RR with 0 points in our table is in the range of 12–20, the same intervals used in the NEWS score, which is validated by Smith et al. [19] and also in patients hospitalized with COVID-19 infection [20]. Hence, we used RR 9–11 with 1 point due to a lower respiratory rate than normal, which might have been due to different causes. The original MEWS [3] had the RR interval of 9–14 with 0 points, so to assess if there is any difference between MEWS with other vital sign ranges and to not leave any stone unturned, we reanalyzed the data with the following results. For the MEWS score used in our study, the best cutoff point is ≥6, with an accuracy of 0.667, sensitivity of 0.675, specificity of 0.646, and AUC of 0.731. For the original MEWS, the best cutoff point is ≥6 with an accuracy of 0.576, sensitivity of 0.463, specificity of 0.872, and AUC of 0.736. The assessed parameters did not change a lot, with no impact on the final cutoff point or conclusions. It is thus possible that in the future, these scores might change, with intervals becoming even smaller, so patients might gain points very quickly, which could benefit them due to earlier evaluation from an ICU physician.

## 5. Conclusions

Further cohorts are needed to validate these scores in other hematologic disease treating centers and the suggested cutoff points. A simple scoring system such as the MEWS can provide an opportunity to provide a prognosis for a patient with hematological disease admitted to the ICU. It also has to be noted that the scores and cutoff points were determined on a sample of hematologic patients that were already admitted to the ICU. Thus, an extension of these scores outside hematologic patients or considering hematologic patients outside the ICU has to be further studied. As a future perspective, we intend to study these scores on hematology ward patients and determine which scores and cutoffs should be used to send these patients to the ICU while still being manageable. The final goal is to encourage physicians to use these scores to improve the early recognition of organ failure to admit patients to the ICU sooner and improve the overall chances of survival.

## Figures and Tables

**Table 1:** MEWS used in our study.

MEWS	3	2	1	0	1	2	3
Respiratory rate (breaths/min)	≤8		9–11	12–20		21–24	≥25
Temperature (°C)		>38.9	38–38.9	36–37.9	35–35.9	34–34.9	<34
Systolic BP (mmHg)	≤90	91–100	101–110	111–199		>199–219	≥220
Heart rate (bpm)	≤40		41–50	51–90	91–110	111–129	≥130
GCS				14–15	10–13	4–9	3
Urine production (mL/h)	0	<20	<35		>200		

GCS: Glasgow Coma Scale/Score; MEWS: The Modified Early Warning Score.

**Table 2:** General characteristics of the cohort. ICU = intensive care unit; MM = multiple myeloma; AL = acute leukemia, CLL = chronic lymphocytic leukemia; MDS = myelodysplastic syndrome; MPN = myeloproliferative neoplasm; CML = chronic myelogenous leukemia. The values in brackets represent percentages and IQR.

Variable		Overall, *n* = 174	Dead, *n* = 126	Alive, *n* = 48	*p* Value
Male		92 (52.9%)	62 (49.2%)	30 (62.5%)	0.129
Age (years)		62 (49, 69)	63 (50, 66)	60 (48, 66)	0.215
Days in ICU		4 (1, 7)	3 (1, 6)	6 (2, 9)	<0.01
Days in hospital before ICU		5 (1, 14)	7 (1, 14)	4 (1, 13)	0.332
Diagnosis	AL	65 (37.8%)	53 (42.1%)	12 (25.0%)	0.397
	CLL	16 (9.3%)	9 (7.1%)	7 (14.6%)	
	CML	2 (1.2%)	1 (0.8%)	1 (2.1%)	
	Lymphoma	41 (23.8%)	29 (23.0%)	12 (25.0%)	
	MDS	8 (4.7%)	5 (4.0%)	3 (6.3%)	
	MM	27 (15.7%)	19 (15.1%)	8 (16.7%)	
	MPN	4 (2.3%)	3 (2.4%)	1 (2.1%)	
	Other	9 (5.2%)	6 (4.8%)	3 (6.3%)	
Chemotherapy protocol	Intensive	74 (42.5%)	53 (42.1%)	21 (43.8%)	0.109
	Non-intensive	61 (35.1%)	40 (31.7%)	21 (43.8%)	
	Unknown	39 (22.4%)	33 (26.2%)	6 (12.5%)	

## Data Availability

All data is available, either analyzed as figures and tables presented in the current manuscript; or as raw data upon request by any external collaborator or reviewer.
